# Defensive Behaviors Driven by a Hypothalamic-Ventral Midbrain Circuit

**DOI:** 10.1523/ENEURO.0156-19.2019

**Published:** 2019-07-26

**Authors:** Leandra R. Mangieri, Zhiying Jiang, Yungang Lu, Yuanzhong Xu, Ryan M. Cassidy, Nicholas Justice, Yong Xu, Benjamin R. Arenkiel, Qingchun Tong

**Affiliations:** 1 Brown Foundation Institute of Molecular Medicine of McGovern Medical School, University of Texas Health Science Center at Houston, Houston, TX 77030; 2 Graduate Program in Neuroscience of MD Anderson Cancer Center UTHealth Graduate School of Biomedical Sciences, University of Texas Health Science Center at Houston, Houston, TX 77030; 3 Department of Neurobiology and Anatomy of McGovern Medical School, University of Texas Health Science Center at Houston, Houston, TX 77030; 4 Children’s Nutrition Research Center, Department of Pediatrics, Baylor College of Medicine, Houston, TX 77030; 5 Department of Neuroscience, Baylor College of Medicine, and Jan and Dan Duncan Neurological Research Institute, Texas Children’s Hospital, Houston, TX 77030

**Keywords:** feeding, glutamate, grooming, jumping, midbrain, PVH

## Abstract

The paraventricular hypothalamus (PVH) regulates stress, feeding behaviors and other homeostatic processes, but whether PVH also drives defensive states remains unknown. Here we showed that photostimulation of PVH neurons in mice elicited escape jumping, a typical defensive behavior. We mapped PVH outputs that densely terminate in the ventral midbrain (vMB) area, and found that activation of the PVH→vMB circuit produced profound defensive behavioral changes, including escape jumping, hiding, hyperlocomotion, and learned aversion. Electrophysiological recordings showed excitatory postsynaptic input onto vMB neurons via PVH fiber activation, and *in vivo* studies demonstrated that glutamate transmission from PVH→vMB was required for the evoked behavioral responses. Photostimulation of PVH→vMB fibers induced cFos expression mainly in non-dopaminergic neurons. Using a dual optogenetic-chemogenetic strategy, we further revealed that escape jumping and hiding were partially contributed by the activation of midbrain glutamatergic neurons. Taken together, our work unveils a hypothalamic-vMB circuit that encodes defensive properties, which may be implicated in stress-induced defensive responses.

## Significance Statement

Paraventricular hypothalamus (PVH) neurons are known to be involved in various homeostatic processes. Despite the known activation of PVH neurons by various stressors, whether these neurons are directly involved in defensive behaviors during stressful events is not clear. This study reveals a direction projection from PVH to ventral midbrain (vMB) region. Acute activation of either PVH neurons or specific PVH→vMB projections elicited defensive-related behaviors, including escape jumping, hiding and learned aversion, which was partially contributed by midbrain glutamatergic neurons. Our study thus identifies a previously unknown role for the PVH→vMB neural pathway in promoting a defensive behavioral program.

## Introduction

Defensive behaviors encompass a repertoire of hard-wired responses critical for survival in the animal kingdom (Blanchard and Blanchard, 2008). Perceived threats prompt expression of fear, and result in escape behaviors, such as fleeing or freezing (Steimer, 2002). Such behaviors are orchestrated by intricate neural networks, comprising multiple brain sites and likely redundant pathways (Silva et al., 2016a). The hypothalamus is a complex structure that contains spatially distinct groups of neurons with diverse functions. In addition to its well-established role in homeostatic processes via endocrine or autonomic control, the ventromedial hypothalamus (VMH) is also implicated in innate defensive responses (Wang et al., 2015), as well as the associated emotional states and learned responses to threat (Kunwar et al., 2015; Silva et al., 2016b). Whether other hypothalamic neurons are also involved in innate behaviors is not clear.

The paraventricular hypothalamus (PVH) has been classically described as a central hub for an array of autonomic and neuroendocrine functions essential for homeostasis (Ferguson et al., 2008), and as a key output node for adapting internal metabolic activity to energy status (Sutton et al., 2016). We have recently shown that the activity level of PVH neurons dictates feeding versus stress-related self-grooming, providing evidence that PVH may integrate information across several modalities to adjust emotional and behavioral output (Mangieri et al., 2018). Indeed, recent studies suggest a role for PVH neurons in mediating behavioral aspects of the stress response (Füzesi et al., 2016). Given that encountering various stressors is an integral part of ensuing changes in emotional states and behavior, it is possible that PVH neurons are involved in these processes. PVH neurons project to mesolimbic structures such as midbrain regions within and surrounding the ventral tegmental area (VTA; Geerling et al., 2010; Watabe-Uchida et al., 2012), and specific oxytocin projection to the VTA regulates pro-social behavior (Hung et al., 2017). Of note, previous studies describe motivational and behavioral changes, including elicitation of defensive behaviors, following electrical stimulation of broad PVH area (Atrens and Von, 1972; Lammers et al., 1987, 1988). However, whether PVH neurons directly drive defensive behaviors is unknown.

The VTA and nearby regions of the midbrain [thereafter referred to as ventral midbrain (vMB)] are composed of heterogeneous neuron populations, including dopaminergic, GABAergic and glutamatergic neurons (Morales and Margolis, 2017). Dopamine neurons are well known for driving reward, a positive emotional state, while glutamatergic neurons have recently been shown to drive aversion (Morales and Margolis, 2017), a negative emotional state associated with fear and anxiety. Here, we uncover a pathway from PVH to the vMB region that drives innate defensive behaviors, including escape, learned avoidance, and feeding suppression, some of which were partially mediated by midbrain glutamatergic neurons. Collectively, these findings suggest that the PVH→vMB projection represents a novel component of defensive neurocircuitry, and provide a potential link between negative emotions (stress and fear) and feeding abnormalities.

## Materials and Methods

### Animals

Animal care and procedures were approved by the University of Texas Health Science Center at Houston Institutional Animal Care and Use Committee. Mice were housed at 21–22°C on a 12/12 h light/dark cycle (7 A.M. to 7 P.M. light), with *ad libitum* access to standard pellet chow, unless otherwise stated during fasting experiments. Sim1-Cre mice (Balthasar et al., 2005) were bred to Ai9 reporter mice (Madisen et al., 2010) to generate Sim1-Cre::Ai9; some of the subjects used in behavioral experiments contained the reporter gene for post-hoc visualization purposes. Sim1-Cre::Vglut2^F/F^ mice were generated as previously described (Xu et al., 2013). Vglut2-ires-Cre mice (Vong et al., 2011) were purchased from The Jackson Laboratory (stock no. 016963) and bred to C57 mice to generate Vglut2-ires-Cre subjects used in the experiments. Mice were at least six weeks old before surgeries and testing, and were chosen from multiple litters. All experiments were done in males during the light cycle, between the early afternoon hours (12 P.M.) and early evening before the start of the dark cycle.

### Viruses and surgery

The following viral constructs were delivered to the PVH via stereotactic surgery. For optogenetic experiments, AAV-EF1α-DIO-hChR2(H134R)-EYFP-WPRE-hGHpA serotype 2/9 (IDDRC Neuroconnectivity Core, Baylor College of Medicine, Houston, TX); AAV-EF1α-DIO-EGFP serotype DJ8 (IDDRC Neuroconnectivity Core); AAV-EF1α-DIO-iC++-EYFP (University of North Carolina Vector Core, Chapel Hill, NC). For anterograde tracing, AAV-EF1α-FLEX-Syn-EGFP-WPRE-hGHpA, serotype DJ/8 (IDDRC Neuroconnectivity Core). For *ex vivo* electrophysiological recordings of Vglut2 positive and negative neurons in the midbrain, channelrhodopsin-2 (ChR2) virus as above was injected to PVH and AAV-EF1α-DIO-EGFP serotype DJ8 virus was injected to the midbrain to label Vglut2-positive cells. For combined optogenetic/DREADD-mediated inhibition, ChR2 virus as above was injected to PVH and AAV1-EF1α-DIO-hM4D(Gi)-mCherry EYFP (University of North Carolina Vector Core) was injected in the midbrain-VTA area. For fiber photometry experiments, AAV-EF1α-FLEX-GCaMP6m (IDDRC Neuroconnectivity Core) was delivered to the midbrain-VTA area. All viral preparations were tittered to at least 10^11^ particles/ml.

Stereotaxic surgeries to deliver viral constructs and for optical fiber implantation were performed as previously described (Mangieri et al., 2018). Briefly, mice were anesthetized with a ketamine/xylazine cocktail (100 and 10 mg/kg, respectively), and their heads affixed to a stereotaxic apparatus. Viral vectors were delivered through a 0.5-µl syringe (Neuros Model 7000.5 KH, point style 3; Hamilton) mounted on a motorized stereotaxic injector (Quintessential Stereotaxic Injector; Stoelting) at a rate of 40 nl/min. Viral delivery was targeted to the PVH (100 nl/side; AP: −0.5 mm; ML: ±0.2 mm; DV: −5.0 mm) or midbrain/VTA area (200–300 nl/side AP: −2.4 mm; ML: ±0.5  mm; DV: −4.6 mm). Uncleaved fiber optic cannulae (Ø1.25-mm stainless ferrule, Ø200-µm core, 0.39 NA; ThorLabs) were precut to 4.5–4.8 mm and implanted above the PVH (AP: −0.5 mm; ML: 0 mm) or precut to 4.3–4.5 mm and implanted above midbrain/VTA (AP: –2.4 mm; ML: +0.5 mm). For glutamate receptor blockade experiments, a single cannula system allowing for interchangeable optic fiber and fluid delivery (Plastics1) was implanted above the midbrain/VTA area. For fiber photometry, uncleaved fiber optic cannulae (Ø1.25-mm stainless ferrule, Ø400-µm core, 0.39 NA; ThorLabs) were precut to 4.3–4.5 mm and implanted above the midbrain/VTA area. All cannulae implants were secured on the head with adhesive gel (Loctite 454) and dental cement. Experiments were conducted on subjects after a three- to four-week recovery period following surgery.

### Acute brain slices preparation and *in vitro* electrophysiology recordings

For Sim1-Cre mouse recordings, coronal brain slices (250–300 μm) containing the PVH or VTA from mice that had received stereotaxic injections of AAV-FLEX-ChR2-EYFP to PVH at least three weeks before the recording were cut in ice-cold artificial CSF (aCSF) containing the following the following: 125 mM NaCl, 2.5 mM KCl, 1 mM MgCl_2_, 2 mM CaCl_2_, 1.25 mM NaH_2_PO_4_, 25 mM NaHCO_3_, and 11 mM D-glucose bubbling with 95% O_2_/5% CO_2_. Slices containing the PVH were immediately transferred to a holding chamber and submerged in oxygenated aCSF. Slices were maintained for recovery for at least 1 h at 32–34°C before transferring to a recording chamber. Individual slices were transferred to a recording chamber mounted on an upright microscope (Olympus BX51WI) and continuously superfused (2 ml/min) with aCSF warmed to 32–34°C by passing it through a feedback-controlled in-line heater (TC-324B; Warner Instruments). Cells were visualized through a 40× water-immersion objective with differential interference contrast (DIC) optics and infrared illumination. Whole-cell current-clamp recordings were made from neurons within the regions of the PVH showing high density of ChR2-EYFP expression, and whole-cell voltage-clamp recordings in VTA/midbrain region were performed on cells surrounded by dense ChR2-EYFP expressing fibers. Pipettes were filled with a K^+^-based low Cl^−^ internal solution containing the following: 145 mM KGlu, 10 mM HEPES, 0.2 mM EGTA, 1 mM MgCl_2_, 4 mM Mg-ATP, 0.3 mM Na_2_-GTP, and 10 mM Na_2_-phosphocreatine (pH 7.3 adjusted with KOH; 295 mOsm) for current clamp recordings. For voltage-clamp recordings, Patch pipettes (3–5 MΩ) were filled with a Cs^+^-based low Cl^−^ internal solution containing the following: 135 mM CsMeSO_3_, 10 mM HEPES, 1 mM EGTA, 3.3 mM QX-314, 4 mM Mg-ATP, 0.3 mM Na_2_-GTP, and 8 mM Na_2_-phosphocreatine (pH 7.3 adjusted with CsOH; 295 mOsm). Membrane potentials were corrected for ∼10 mV liquid junction potential. To activate ChR2-expressing neurons in PVH or CHR2-fibers in VTA/midbrain, light from a 473-nm laser (Opto Engine LLC) was focused on the area of the recorded PVH neuron to produce spot illumination through optic fiber. Brief pulses of light (blue light, 1–2 ms, 1–2 mW/mm^2^) were delivered at the recording site at 10- to 15-s intervals under control of the acquisition software.

Vglut2-ires-cre mice, at least three weeks following virus infection, were anesthetized and brains were obtained for recording. Horizontal slices (250 µm) containing the VTA were sectioned using a Leica VT 1000S vibratome, and transferred to a holding chamber with aCSF containing the following: 123 mM NaCl, 26 mM NaHCO_3_, 2.5 mM KCl, 1.25 mM NaH_2_PO_4_, 10 mM glucose, 1.3 mM MgCl_2_, and 2.5 mM CaCl_2_ and saturated with 95% O_2_/5% CO_2_ at 32°C for 1 h, then maintained at room temperature to allow for recovery before any electrophysiological recordings. Individual slices were transferred from the holding chamber to a heated recording chamber (31–33°C, Luigs-Neumann), in which they were submerged and continuously perfused with oxygenated aCSF at a rate of 2–3 ml/min. Recordings were performed under infrared-DIC visualization on a fixed stage, upright microscope (Olympus BX51WI) equipped with a water immersion 40× objective. Pipettes with resistance 3–5 MΩ were pulled from borosilicate glass (OD 1.5 mm, ID 1.1 mm; Sutter Instruments) using a horizontal puller (Sutter P-1000), and filled with an internal patch solution containing the following: 142 mM K-gluconate, 10 mM HEPES, 1 mM EGTA, 2.5 mM MgCl_2_, 0.25 mM CaCl_2_, 4 mM Mg-ATP, 0.3 mM Na-GTP, and 10 mM Na_2_-phosphocreatine, adjusted to pH 7.25–7.35, osmolality 295–305 with KOH. Whole-cell patch-clamp recordings data were digitized and collected using Multiclamp 700B amplifier, and Digidata 1550B digitizer, and Clampex 10 (Molecular Devices). Membrane potential was held at –60 mV. The liquid junction potential was not corrected, and series resistance (Rs) was bridge balanced. Offline data analysis was performed using Clampfit 10 (Molecular Devices). To excite ChR in brain slices, we illuminated the brain slices every 30s with blue light pulses (473-nm PSU-III-LED laser system, Optoengine), of short duration (1–3 ms) through 40× water-immersion objective lens.

### Optogenetic experimental parameters

For *in vivo* photostimulation/inhibition, an integrated rotary joint patch cable connected the ferrule end of optic fiber cannula with a Ø1.25 mm ferrule end of the patch cable via a mating ceramic sleeve (ThorLabs). At the other end of the rotary joint, an FC/PC connector was connected to a 473-nm diode-pumped solid state (DPSS) laser (Opto Engine LLC). Light pulses were controlled by Master-8 pulse stimulator (A.M.P.I.). For behavioral experiments requiring a large chamber [real-time place preference/avoidance (RTPP/A), locomotion, and escape hut assays] a commutator (rotary joint; Doric) was attached to a patch cable via FC/PC adapter. The patch cable was then attached to the optic fiber cannula ferrule end via a ceramic mating sleeve. Another patch cable containing FC/PC connections at both ends allowed the connection between the commutator and the laser, which was controlled by the Master-8 pulse stimulator. During testing, mice were placed in a clean, high-walled enclosure or in a large chamber wiped down with 70% isopropyl alcohol. Light power was measured before starting experiments each day with an optical power meter (ThorLabs), and adjusted to emit an output of 5–15 mW from the end of the mating sleeve.

### Behavioral analysis

#### Grooming and escape jumping

To measure the effects of photostimulation on the baseline behavior, mice were placed in a clean, high-walled enclosure, which prevented escape from the chamber. Sim1-Cre mice were observed for grooming and recorded with a hand-held camera for a 15-min period with the following protocol: 5 min, no light (pre), 5 min, light-on (on), and 5 min post-light (post). A 6-min observation period for jumping behavior in Sim1-Cre mice was performed following 2-min pre-light, 2-min light-on, and 2-min post-light. Vglut2-ires-cre mice were observed similarly for grooming and jumping during the 15-min protocol.

For PVH photostimulation, light was pulsed at a 5-Hz frequency with 10- or 100-ms pulse duration, and 20 Hz 10 ms for PVH→vMB photostimulation. Behavioral changes were annotated by watching the videos using QuickTime Player (Apple). Time spent grooming was carefully annotated by noting the video timestamps at the beginning and end of grooming bouts. Beginning of bouts was defined as the moment the animal started engaging in forelimb paw strokes made near the nose, eyes, and head, and licking of paw, body, tail, or genitals, and the end of bouts was noted when grooming was interrupted for at least 6 s. The latency to start grooming was defined as the precise time mice started grooming following the first pulse of light. Number of jumps during the 15-min test was quantified by watching videos in slow motion and counting each jump mice made, as defined by removal of limbs from the floor of the cage and complete suspension of the body in air. Grooming and escape jumping observations were also performed 1 h following intraperitoneal injection of saline or clozapine-N-oxide (CNO; 1 mg/kg) in Vglut2-ires-Cre mice expressing hM4D(Gi)-mCherry in the vMB.

#### Glutamate receptor blockade

Mice implanted with interchangeable fluid delivery/optic fiber cannula system (Plastics1) were anesthetized with isoflurane and placed in a stereotactic apparatus. A microinjection volume of 100 nl, directed to the midbrain/VTA area, was slowly infused at an approximate rate of 33 nl/min. Three minutes following infusion, fluid delivery cannulae were removed from the guiding cannula and replaced with optic fiber cannula, and mice were allowed to recover from anesthesia for 10–15 min before testing. Mice were then placed in a high-walled enclosure and video recorded for 5 min during photostimulation (20-Hz 10-ms pulses). Jumping behavior was annotated as described above. Two separate trials were performed at the same mice on separate days: a control (vehicle injection) trial and drug (glutamate receptor blockade injection) trial. Vehicle injections consisted of 15% DMSO, while drug injections consisted of 300-ng D-AP5 + 150-ng DNQX (Tocris) suspended in 15% DMSO.

#### Locomotion

Mice were placed in a large (45 × 45 × 50 cm^3^) chamber, equipped with an overhead infrared camera (PhenoTyper system 3.0, Noldus), and allowed to freely roam during a 15-min test, consisting of 5-min no-light, 5-min light-on, and 5-min post-light. Light was pulsed at 5 Hz 10 ms for PVH photostimulation, and 20 Hz 10 ms for PVH→vMB photostimulation. Locomotion assays were also performed 1 h following intraperitoneal injection of saline or CNO (1 mg/kg) in Vglut2-ires-Cre mice expressing hM4D(Gi)-mCherry in the vMB. Locomotion data, including total distance traveled and average velocity, were collected by tracking software (EthoVision XT 11.5, Noldus) for each 5-min period. Activity tracks were visualized by plotting movement of the mouse based on center point location, as captured by the overhead camera.

#### RTPP/A assays

For RTPP/A assays, mice were allowed to freely explore a large 45 × 45 × 50 cm^3^ chamber, as detailed above, during a 20-min testing period. The chamber was evenly divided into two sectors, one of which was randomly assigned as the light-on side. Crossing over and occupying the light-paired side of the chamber triggered continuous pulsing of light (5-Hz 100- ms light pulses for PVH photostimulation, and 20-Hz 10-ms pulses for PVH→vMB photostimulation), which ceased once animals returned to the light-off side. The side of the chamber paired with light was counterbalanced during experiments for each mouse. RTPP/A assays were performed 1 h following intraperitoneal injection of saline or CNO (1 mg/kg) in Vglut2-ires-Cre mice expressing hM4D(Gi)-mCherry in the midbrain. The percentage time spent on each side and time spent in the food zone, as well as the tracking data, were collected by EthoVision tracking software (Noldus). Heatmaps detailing proportion of time spent in each location of the arena, as well as activity tracks, were visualized based on the data collected.

#### Modified RTPP/A assay-fast refeed

Mice were fasted 24 h before testing fast-refeeding in a large chamber containing food in one corner of the arena. The location of food was rotated among four corners of the cage, and the light-paired side was counterbalanced for each mouse tested. On crossing into the light-paired side, light was pulsed through the optical fiber into the brain at 5 Hz 10 ms for PVH activation or 20 Hz 10 ms for PVH→vMB activation, and ceased on exit into the light-unpaired side. Total testing time lasted 15 min. The percentage time spent on each side and time spent in the food zone, as well as the tracking data, were collected by EthoVision tracking software (Noldus). Heatmaps detailing proportion of time spent in each location of the arena, as well as activity tracks, were visualized based on the data collected.

#### Conditioning assay

Sim1-Cre mice with ChR2 injected into the PVH and optical fibers placed over the vMB, were placed in a large testing chamber with flooring on one side lined with several columns of green tape spanning the top to bottom edges of the cage. On day 0, mice tethered to an optic fiber cable delivering no light, were allowed to freely explore the arena for 20 min; the side most preferred, as determined by percentage time spent on each side, was noted and assigned as the light-paired side for the subsequent days of conditioning. For the next 4 d of conditioning, mice were tested approximately at the same time for 20 min, during which optic fiber cable delivered 20-Hz 10-ms photostimulation on crossing the light-paired side of the chamber, and ceased once mice traversed to the light-off side. Mice were thereafter tested for 20 min on days 5–6 for extinction, during which light was no longer delivered through the optic fiber. The preference for the light-on zone, initially the most preferred side, was calculated as the percentage time mice spent in the light-paired side of the chamber for each trial. Locomotion data to calculate the distance traveled during testing sessions were collected by EthoVision tracking software (Noldus).

#### Escape hut assay

For this assay, an “escape hut,” equipped with a single entry and three 9.5-cm walls with no “roof” (to maintain top-down visualization of tracking from the overhead camera) was placed in the center of a large chamber. Testing was performed 1 h following intraperitoneal injection of saline or CNO (1 mg/kg) in Vglut2-ires-Cre mice expressing hM4D(Gi)-mCherry in vMB. Mice were first acclimated to the novel environment for 7 min, which allowed sufficient time for spontaneous discovery of the hut. After acclimation, an 8-min testing period immediately followed, in which light was continuously pulsed at 5–10 Hz (10-ms pulse width) every other minute. The number of hut visits (defined as the number of times the animal approached and entered the hut) and duration in the hut (quantified as the time spent inside the hut enclosure) were quantified by EthoVision software (Noldus). Number of hut visits and total time spent inside the hut across each time interval (off vs on) was combined for statistical analysis. Total distance traveled during each time interval was also combined for analysis, and velocity was averaged across each light-off and light-on periods to reveal average velocity during the two light conditions. Heatmaps across time intervals were constructed based on tracking data collected by EthoVision software.

### Immunohistochemistry (IHC) and imaging

After behavioral experiments were completed, study subjects were anesthetized with a ketamine/xylazine cocktail (100 and 10 mg/kg, respectively) and subjected to transcardial perfusion. Freshly fixed brains were then extracted and placed in 10% buffered formalin at 4°C overnight for post-fixation. The next day, brains were transferred to 30% sucrose solution and allowed to rock at room temperature for 24 h before sectioning. Brains were frozen and sectioned into 30 µm slices with a sliding microtome and mounted onto slides for post-hoc visualization of injection sites and cannula placements. Injection sites were determined by the densest regions of EYFP, EGFP, or mCherry fusion products. The location of cannula implants was noted by prominent lesion sites that extended over the rostro-caudal axis of the PVH or the vMB area. Mice with missed injections to the PVH or vMB, or those with misplaced optic fibers over the areas of interest were excluded from behavioral analysis. Representative pictures of PVH, PVH projections, and vMB injection sites were visualized with confocal microscopy (Leica TCS SP5; Leica Microsystems). Brain sections used for IHC were stained with the following primary antibodies, followed by secondary antibodies: mouse anti-tyrosine hydroxylase (TH; Millipore, MAB318)/Alexa Fluor 488, donkey anti-mouse or Alexa Fluor 594 donkey anti-mouse; rabbit anti-cFos (Cell Signal #2250)/Alexa Fluor 488 donkey anti-rabbit, or Alexa Fluor 594 donkey anti-rabbit. Sim1-Cre mice used for cFos analysis were placed separately in clean testing cages, provided with food, water, and bedding for 2 h before photostimulation. Mice were then photostimulated with 20-Hz light pulses (10-ms pulse duration) for 5 s, followed by 5 s of no light, repeated for 15 min. Following photostimulation, mice were subjected to transcardial perfusion 1.5 h later, and brain sections were processed for IHC. Vglut2-ires-Cre mice used for cFos analysis were first intraperitoneally injected with CNO, and placed in clean testing cages 30 min before photostimulation. Photostimulation was then applied for 15 min (20-Hz 10-ms pulses every 5 s), and mice were transcardially perfused 1.5 h later.

### Statistics

GraphPad Prism 7 software was used for statistical analysis. Two-way repeated measures ANOVA, followed by Dunnett’s or Sidak’s multiple comparisons tests, and ordinary or repeated measures one-way ANOVA tests, followed by Dunnett’s or Tukey’s multiple comparisons test, were used for comparisons of more than two groups. Paired or unpaired two-tailed *t* tests were used for comparing two groups. Pearson correlation (two-tailed) was used to analyze correlation between two variables. Data in figures, text, and legends were expressed as mean ± SEM. Significance levels were denoted by asterisks: *p < 0.05, **p < 0.01, ***p < 0.001.

## Results

### Activation of PVH neurons elicits escape behavior associated with increased flight and negative valence

**Figure 1.**
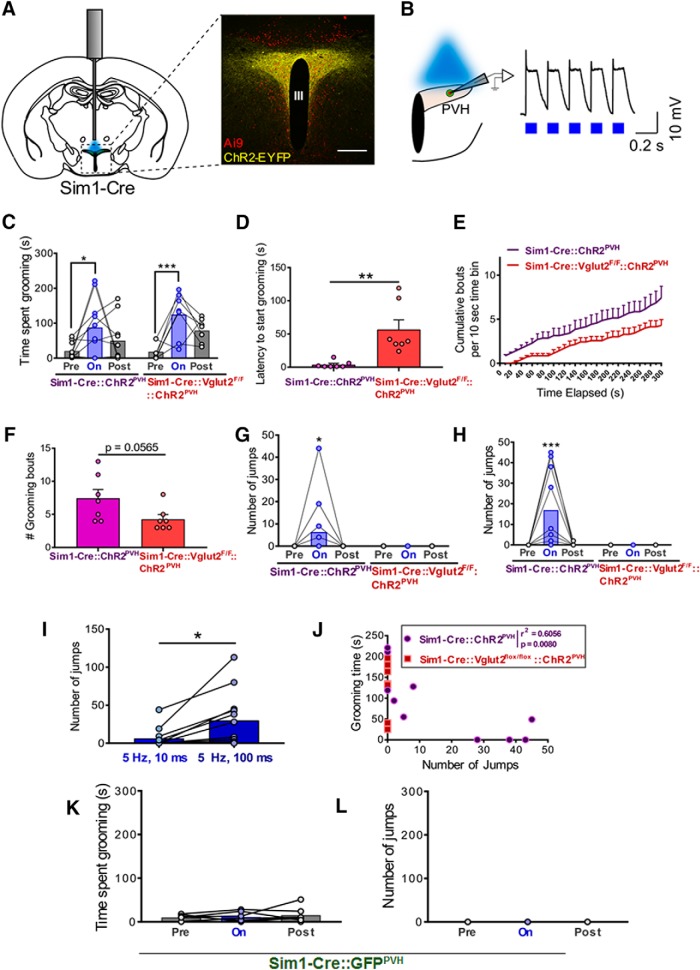
Optogenetic activation of PVH neurons elicits flight and rscape behaviors. ***A***, Experimental schematic (left) and ChR2-EYFP expression in Sim1-Cre::Ai9 neurons in PVH (right). III, third ventricle. Scale bar, 300 µm. ***B***, Whole-cell recordings in PVH-ChR2 neurons responding to 5-Hz 100-ms light pulses. Quantification of time spent grooming in live animals during 5-Hz 100-ms photostimulation of PVH; *n* = 7–10 animals/group. Two-way repeated measures ANOVA, followed by Dunnett’s multiple comparisons test: interaction *F*_(2,30)_ = 0.6948, *p* = 0.5070; genotype *F*_(1,15)_ = 1.316, *p* = 0.2692; light epoch *F*_(2,30)_ = 12.33, *p* = 0.0001; subjects (matching) *F*_(15,30)_ = 1.699, *p* = 0.1056. Pre-light versus light-on, *p < 0.05, ***p < 0.0005. ***C***, Time spent grooming before, during, and after 5-Hz 100-ms photostimulation of PVH in Sim1-Cre::GFP^PVH^ control mice; *n* = 7 animals. One-way repeated measures ANOVA: light epoch *F*_(1.619,9.715)_ = 0.5018, *p* = 0.5827; animals *F*_(6,12)_ = 2.2, *p* = 0.1155. ***D***, Latency to initiate grooming following the first pulse of light during a 5-min PVH-photostimulation session; *n* = 7 animals/group. Unpaired *t* test: *t* = 3.657 df = 12; **p = 0.0033. Error bars represent SEM. ***E***, Temporal representation of cumulative grooming bouts, calculated every 10 s, during 5 min of photostimulation; *n* = 7 animals/group. Error bars represent SEM. ***F***, Comparison of total number of grooming bouts between Sim1-Cre and Sim1-Cre::Vglut2^F/F^ animals during the 5 min of photostimulation; *n* = 7 animals/group. Unpaired *t* test: *t* = 2.11 df = 12; *p* = 0.0565. Error bars represent SEM. ***G***, Number of jumps counted during 5 min of PVH photostimulation with 5-Hz 10-ms pulses of light; *n* = 7–12 animals/group. Two-way repeated measures ANOVA, followed by Dunnett’s multiple comparisons test: interaction *F*_(2,34)_ = 1.576, *p* = 0.2215; genotype *F*_(1,17)_ = 1.576, *p* = 0.2263; light epoch *F*_(2,34)_ = 1.576, *p* = 0.2215; subjects (matching) *F*_(17,34)_ = 1, *p* = 0.4813. Pre-light versus light-on, *p < 0.05. ***H***, Number of jumps elicited by 5-Hz 100-ms photostimulation of PVH; *n* = 7–10 animals/group. Two-way repeated measures ANOVA, followed by Dunnett’s multiple comparisons test: interaction *F*_(2,30)_ = 5.299, *p* = 0.0107; genotype *F*_(1,15)_ = 5.24, *p* = 0.0370; light epoch *F*_(2,30)_ = 5.299, *p* = 0.0107; subjects (matching) *F*_(15,30)_ = 1.048, *p* = 0.4392. Pre-light versus light-on, ***p < 0.0005. ***I***, Comparison of the number of jumps evoked by 5 min of 5-Hz 10- or 100-ms photostimulation in Sim1-Cre mice; *n* = 12 animals. Paired *t* test: *t* = 2.965 df = 11; *p = 0.0129. Error bars represent SEM. ***J***, Correlation between grooming time and number of jumps in Sim1-Cre animals during 5-Hz 100-ms PVH photostimulation; *n* = 7–10 animals/group. Pearson correlation: *r* ^2^ = 0.6056, *p* = 0.0080. ***K***, Time spent in grooming evoked by 5 min of laser stimulation in Sim1-Cre::GFP^PVH^ control mice (*n* = 7). ***L***, Number of jumps evoked by 5 min of laser stimulation in Sim1-Cre::GFP^PVH^ control mice (*n* = 7).

Through targeted manipulation of PVH neurons, we recently uncovered a novel hypothalamic site that bidirectionally controls feeding and repetitive, stress-like self-grooming (Mangieri et al., 2018). Here, we aimed to explore and characterize other behavioral responses by manipulating PVH neural activity. To this end, we injected cre-dependent ChR2 viral constructs into the PVH of Sim1-Cre mice (Sim1-Cre::ChR2^PVH^), allowing optogenetic manipulation of the majority of PVH neurons (Balthasar et al., 2005; Fig. 1*A*). Photostimulaton with long pulses of blue light (100 ms) at 5 Hz reliably elicited time-locked activation of PVH neurons (Fig. 1*B*). Similar to our previous findings, *in vivo* photostimulation of PVH neurons at 5 Hz 100 ms produced repetitive self-grooming in the majority of ChR2-expressing mice (Fig. 1*C*). The same photostimulation in Sim1-Cre::ChR2^PVH^::Vglut2^F/F^ mice (also known as knock-outs, KOs), which lacked Vglut2 (required for presynaptic glutamate release) in Sim1-neurons, also showed a robust increase in repetitive grooming time during light-on periods that was not significantly different from that seen in Sim1-Cre::ChR2^PVH^ mice (Fig. 1*C*). However, self-grooming in Sim1-Cre::ChR2^PVH^ mice was more fragmented than in KO mice (Movies 1, 2). Notably, latency to initiate grooming after light illumination was significantly longer in KOs (Fig. 1*D*; Movie 2). We also noted a trend toward fewer grooming bouts in KOs (Fig. 1*E*,*F*). These results suggest that glutamate release, although not absolutely required for, contributes significantly to the light-induced self-grooming. Interestingly, however, we noted that both 5-Hz 10-ms (Fig. 1*G*) and 5-Hz 100-ms (Fig. 1*H*) photostimulation elicited frantic escape-like jumping in the majority of Sim1-Cre::ChR2^PVH^ mice (Movie 3), but not in KOs (Fig. 1*G*,*H*). Notably, the shorter pulse duration (10 ms, 5 Hz) elicited less jumping responses in Sim1-Cre::ChR2^PVH^ mice, and jumping behavior increased in response to the longer length of light-pulses (Fig. 1*I*), indicating scalability of the behavior via strength of neural activation. We also observed that some Sim1-Cre::ChR2^PVH^ mice displayed only grooming or jumping to the exclusion of the other, while others showed a mix of behaviors during the photostimulation session. In fact, we noted a negative correlation between the two behaviors (Fig. 1*J*), consistent with the mutually exclusive nature of such behaviors. Thus, the self-grooming behavior elicited by photostimulation in Sim1-Cre::ChR2^PVH^ mice (Fig. 1*C*) might be underestimated due to conflicting jumping behaviors (Fig. 1*G*). No self-grooming (Fig. 1*K*) or jumping (Fig. 1*L*) was observed in GFP-injected controls (Sim1-Cre::GFP^PVH^), suggesting a specific effect of photostimulating PVH neurons in promoting the behaviors.

Movie 1. Self-grooming behaviors elicited by optogenetic stimulation of PVH neurons in control Sim1-Cre mice. 10.1523/ENEURO.0156-19.2019.movie.1

Movie 2. Self-grooming behaviors elicited by optogenetic stimulation of PVH neurons in control Sim1-Cre::Vglut2^F/F^ mice. 10.1523/ENEURO.0156-19.2019.movie.2

Movie 3. Jumping behaviors elicited by optogenetic stimulation of PVH neurons in control Sim1-Cre mice. 10.1523/ENEURO.0156-19.2019.movie.3

**Figure 2.**
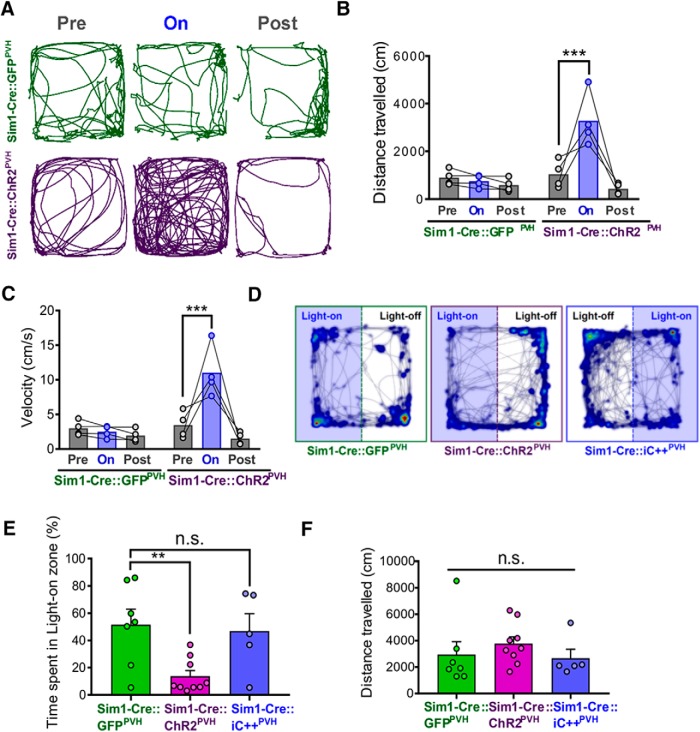
Effects of optogenetic activation of PVH neurons on locomotion and place preference. Behavior observations were made following optogenetic stimulation of PVH Sim1 neurons. ***A***, Representative locomotion traces before, during, and after 5-Hz 10-ms photostimulation of PVH. ***B***, Quantification of distance traveled during locomotion test (***F***); *n* = 4 animals/group. Two-way repeated measures ANOVA, followed by Dunnett’s multiple comparisons test: interaction *F*_(2,12)_ = 12.18, *p* = 0.0013; genotype *F*_(1,6)_ = 16.39, *p* = 0.0067; light epoch *F*_(2,12)_ = 13.47, *p* = 0.0009, subjects (matching) *F*_(6,12)_ = 0.7245, *p* = 0.6385. Pre-light versus light-on, ***p < 0.0005. ***C***, ***H***, Average velocity during the locomotion assay in Figures 1*F*,*G*; *n* = 4 animals/group. Two-way repeated measures ANOVA, followed by Dunnett’s multiple comparisons test: interaction *F*_(2,12)_ = 12.22, *p* = 0.0013; genotype *F*_(1,6)_ = 17.9, *p* = 0.0055; light epoch *F*_(2,12)_ = 13.46, *p* = 0.0009; subjects (matching) *F*_(6,12)_ = 0.6841, *p* = 0.6664. Pre-light versus light-on, ***p < 0.0005. ***D***, Representative heatmaps of time spent in arena location overlaying activity tracks during RTPP/A assay, where one side of the chamber was paired with PVH-photostimulation or inhibition. ***E***, Quantification of percentage time spent in light-on zone during RTPP/A; *n* = 5–9 animals/group. One-way ANOVA, followed by Dunnett’s multiple comparisons test: *F*_(2,18)_ = 6.115, *p* = 0.0094. Sim1-Cre::GFP^PVH^ versus Sim1-Cre::ChR2^PVH^, **p < 0.005. Error bars represent SEM. ***A***, Time spent grooming before, during, and after 5-Hz 100-ms photostimulation of PVH in Sim1-Cre::GFP^PVH^ control mice; *n* = 7 animals. One-way repeated measures ANOVA: light epoch *F*_(1.619,9.715)_ = 0.5018, *p* = 0.5827; animals *F*_(6,12)_ = 2.2, *p* = 0.1155. ***F***, Comparison of distance traveled during the RTPP/A assay in Figures 1*H*,*I*; *n* = 5–9 animals/group. One-way ANOVA: *F*_(2,18)_ = 0.6295, *p* = 0.5442. Error bars represent SEM.

We also found that photostimulation in Sim1-Cre::ChR2^PVH^ mice dramatically increased overall locomotion compared to controls (Fig. 2*A*), affecting both total distance traveled (Fig. 2*B*) and average velocity (Fig. 2*C*), suggesting an elevated state of arousal and agitation. We next probed the emotional valence of PVH activation using an RTPP/A assay (Jennings et al., 2013b). Compared to GFP controls, Sim1-Cre::ChR2^PVH^ mice avoided the light-paired side of the testing chamber, though total distance traveled was unchanged (Fig. 2*D–F*). As an additional comparison, we tested the valence of inhibiting PVH neurons in the RTPP/A assay by using Sim1-Cre mice injected with cre-dependent inhibitory opsin, iC++ (Sim1-Cre::iC++^PVH^; Berndt et al., 2016; Mangieri et al., 2018). Surprisingly, inhibition of PVH neurons did not elicit significant preference or avoidance to the light-paired side (Fig. 2*D–F*), which was previously shown to promote feeding and reduce stress-induced grooming (Mangieri et al., 2018). Collectively, these results indicate that glutamate release from PVH neurons drives a scalable increase in escape behavior, while both glutamate and non-glutamate action contribute to self-grooming.

### PVH projections to the midbrain area drive escape behavior and avoidance

**Figure 3.**
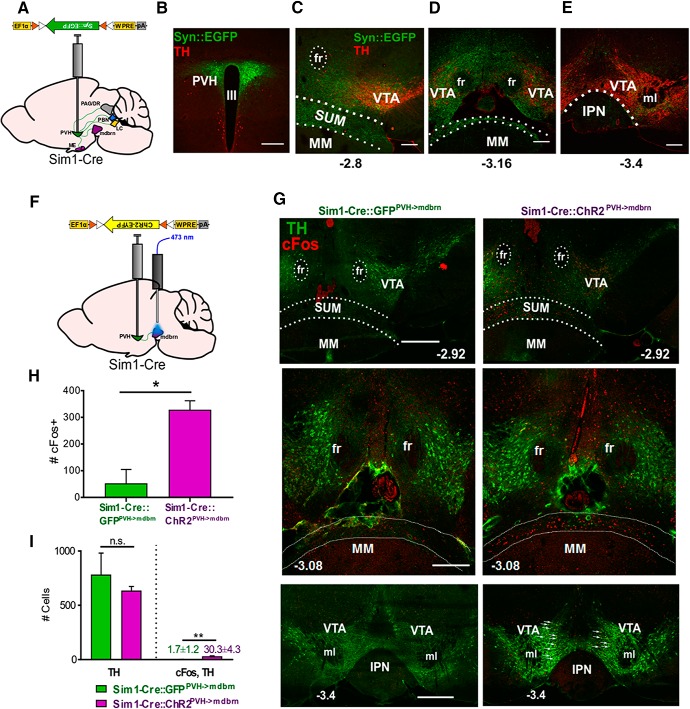
Glutamatergic transmission from PVH to vMB region. ***A***, Anterograde tracing schematic showing downstream sites targeted by PVH projections. ***B***, Synaptophysin-EGFP expression in PVH neurons. III, third ventricle. Scale bar, 300 µm. ***C***, Synaptophysin-EGFP puncta seen in rostral midbrain adjacent to VTA, bregma level –2.8 mm. ***D***, Synaptophysin-EGFP puncta observed in regions surrounding VTA in middle portions of midbrain, bregma level –3.16 mm. ***E***, Synaptophysin-EGFP puncta seen in rostral portions in and surrounding the VTA, bregma level –3.4. fr, fasciculus retroflexus; MM, medial mammillary nucleus. Scale bar, 200 µm. ***F***, Optogenetic activation schematic of PVH→vMB (mdbrn) circuit. ***G***, ***D***, cFos observed in rostral midbrain region (bregma level –2.92 mm, –3.08 mm, and –3.4 mm) adjacent to VTA (indicated by TH immunostaining in green) following photostimulation of PVH→vMB fibers in ChR2-injected animals (right) vs. control (GFP-injected) animals (left). fr, fasciculus retroflexus; MM, medial mammillary nucleus. Arrows point to cFos, TH-positive cells. IPN, interpeduncular nucleus; ml, medial lemiscus. Scale bar, 500 µm. ***H***, Quantification of number of neurons showing cFos staining in GFP and ChR2 mice; *n* = 3 animals/group; two matched sections per mouse were used for quantification. Unpaired *t* test: *t* = 4.456 df = 4; *p = 0.0112. Error bars represent SEM. ***I***, Quantification of cFos-TH neuron overlap in PVH→vMB photostimulated animals. Number of TH neurons was not significantly different between groups, but number of cFos, TH-positive neurons in ChR2 mice was significantly higher versus those found in GFP control mice, though overall number of cFos, TH-positive neurons was low relative to total number of TH cells and cFos-positive neurons (Fig. 2*G*); *n* = 3 animals/group; two matched sections per mouse were used for quantification; *t* test for #TH neurons: *t* = 0.7382, df = 4, *p* = 0.5013, n.s., not significant; *t* test for #cFos, TH neurons: *t* = 6.375, df = 4, **p = 0.0031.

To probe potential PVH targets for the observed behaviors, we injected cre-dependent, synaptophysin constructs (AAV-FLEX-Syn-EGFP) to PVH neurons of Sim1-Cre mice for anterograde tracing (Herman et al., 2016; Figs. 2*B*, 3*A*). We observed dense projections in previously reported sites, such as the median eminence (ME), periaqueductal gray (PAG)/dorsal raphe (DR), parabrachial nucleus (PBN), and locus coeruleus (LC; data not shown). Interestingly, we observed substantial puncta in the midbrain area, both within and surrounding the VTA, most notably in the area medial to VTA and above the mammillary nucleus [supramammillary nucleus (SUM)] and caudally into the VTA area (thereafter called ventral midbrain area (vMB); Fig. 3*C–E*). To evaluate functional connectivity, we photostimulated local Sim1-Cre::ChR2^PVH^ fibers in the vMB (Fig. 3*F*), which evoked time-locked, excitatory postsynaptic currents in vMB neurons in 8 out of 15 neurons recorded, indicating glutamatergic transmission. Following PVH→vMB photostimulation, we found that compared to GFP controls (Sim1-Cre::GFP^PVH->vMB^) mice (Fig. 3*G*, left panels), Sim1-Cre::ChR2^PVH->vMB^ mice (Fig. 3*G*, right panels) had a greater number of cFos-labeled neurons in vMB and SUM (Fig. 3*H*). Most cFos expression was found in the area of the anterior midbrain and medial to the VTA. Notably, few cFos-labeled cells were detected in the VTA region proper, and notably, cFos was found in very few TH+ cells (Fig. 3*I*), consistent with tracing results showing a substantial portion of PVH projections terminating in the SUM and the area medial to the VTA.

**Figure 4.**
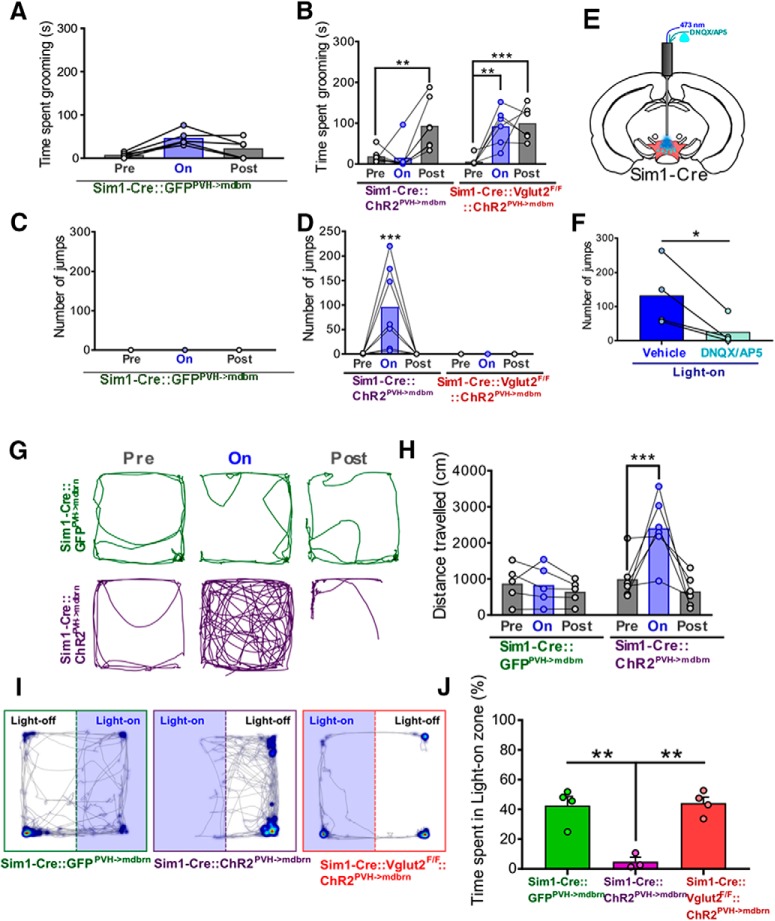
Glutamatergic transmission from PVH to vMB drives flight and escape. ***A***, Time spent grooming in response to 20-Hz 10-ms photostimulation of PVH→vMB (mdbrn) circuit in GFP control mice; *n* = 5 animals. One-way repeated measures ANOVA: light epoch *F*_(1.733,6.931)_ = 11.2, *p* = 0.0077; animals *F*_(4,8)_ = 3.15, *p* = 0.0784. ***B***, Time spent grooming before, during, and after PVH→vMB photostimulation; *n* = 6–7 animals/group. Two-way repeated measures ANOVA, followed by Dunnett’s multiple comparisons test: interaction *F*_(2,22)_ = 5.144, *p* = 0.0147; genotype *F*_(1,11)_ = 2.803, *p* = 0.1223; light epoch *F*_(2,22)_ = 16.47, *p* < 0.0001; subjects (matching) *F*_(11,22)_ = 1.375, *p* = 0.2522. Pre-light versus light-on, **p < 0.005; pre-light versus post-light, **p < 0.005, ***p < 0.0005. ***C***, Number of jumps evoked by 20-Hz 10-ms photostimulation in in GFP control mice; *n* = 5 animals. Mice did not display jumping behavior during any light epoch period. ***D***, Quantification of number of jumps elicited by PVH→vMB photostimulation; *n* = 6–7 animals/group. Two-way repeated measures ANOVA, followed by Dunnett’s multiple comparisons test: interaction *F*_(2,22)_ = 7.756, *p* = 0.0028; genotype *F*_(1,11)_ = 7.69, *p* = 0.0181; light epoch *F*_(2,22)_ = 7.756, *p* = 0.0028; subjects (matching) *F*_(11,22)_ = 1.018, *p* = 0.4636. Pre-light versus light-on, ***p < 0.0005. ***E***, Schematic of pharmacological blockade of glutamate receptors in VTA/vMB area before photostimulation in freely moving animals. ***F***, Number of jumps evoked by PVH→vMB photostimulation following microinjection of vehicle or glutamate receptor antagonists to vMB; *n* = 4 animals. Paired *t* test: *t* = 3.357 df = 3; *p = 0.0438. ***G***, Representative locomotion tracks in response to light activation of PVH→vMB circuit. ***H***, Distance traveled during locomotion assay in ***K***; *n* = 5–6 animals/group. Two-way repeated measures ANOVA, followed by Dunnett’s multiple comparisons test: interaction *F*_(2,18)_ = 7.862, *p* = 0.0035; genotype *F*_(1,9)_ = 5.221, *p* = 0.0482; light epoch *F*_(2,18)_ = 10.31, *p* = 0.0010; subjects (matching) *F*_(9,18)_ = 1.93, *p* = 0.1124. Pre-light versus light-on, ***p < 0.0005. ***I***, Representative heatmaps of time spent in each location superimposed over tracks during RTPP/A assay, where one side of chamber was paired with light activation of PVH→midbrain circuit. ***J***, Quantification of percentage time spent in the light zone during RTPP/A assay; *n* = 3–4 animals/group. One-way ANOVA, followed by Tukey’s multiple comparisons test: *F*_(2,8)_ = 18.84, *p* = 0.0009. Sim1-Cre::GFP^PVH->mdbrn^ versus Sim1-Cre::ChR2^PVH->mdbrn^, **p = 0.0018; Sim1-Cre::ChR2^PVH->mdbrn^ versus Sim1-Cre::Vglut2^flox/flox^::ChR2 ^PVH->mdbrn^, **p = 0.0014. Error bars represent SEM.

Empirically, we found that 20-Hz 10-ms photostimulation of the PVH→midbrain circuit in live Sim1-Cre::ChR2 ^PVH->vMB^ animals resulted in the most obvious behavioral changes, including increased grooming behavior post-stimulation (Fig. 4*B*), and escape-jumping similar to that seen with PVH photostimulation (Fig. 4*D*). The same photostimulation failed to enact obvious repetitive grooming or escape jumping in GFP controls (Fig. 4*A*,*C*). In contrast, Sim1-Cre::ChR2 ^PVH->vMB^::Vglut2^F/F^ (KO) mice exhibited a significant increase in grooming during and after the photostimulation period, but showed no jumping behavior (Fig. 4*B*,*D*). Of note, the effect on self-grooming behavior in Sim1-Cre::ChR2 ^PVH->vMB^ mice might be underestimated owing to competing jumping behavior. We found that microinfused glutamate receptor antagonists to vMB of Sim1-Cre::ChR2 ^PVH->vMB^ mice before photostimulation significantly reduced the escape jumping in response to photostimulation (Fig. 4*E*,*F*), confirming that vMB neurons mediate the behavior. Similar to PVH activation, we also noted that photostimulation of PVH fibers in vMB promoted locomotor activity in Sim1-Cre::ChR2 ^PVH->vMB^ mice (Fig. 4*G*,*H*), but not in GFP controls (Fig. 4*G*,*H*) or KO mice (data not shown), suggesting an essential role for glutamate release in promoting locomotor activity.

Interestingly, we found that place avoidance caused by PVH→vMB photostimulation in the RTPP/A assay required glutamatergic transmission (Fig. 4*I*,*J*), but did not affect the total distance traveled during the assay (data not shown). These results suggest that glutamatergic transmission from PVH→vMB promotes a state of negative valence, coupled by a drive for flight and escape.

### Activation of PVH→vMB circuit suppresses food intake and promotes aversion learning

**Figure 5.**
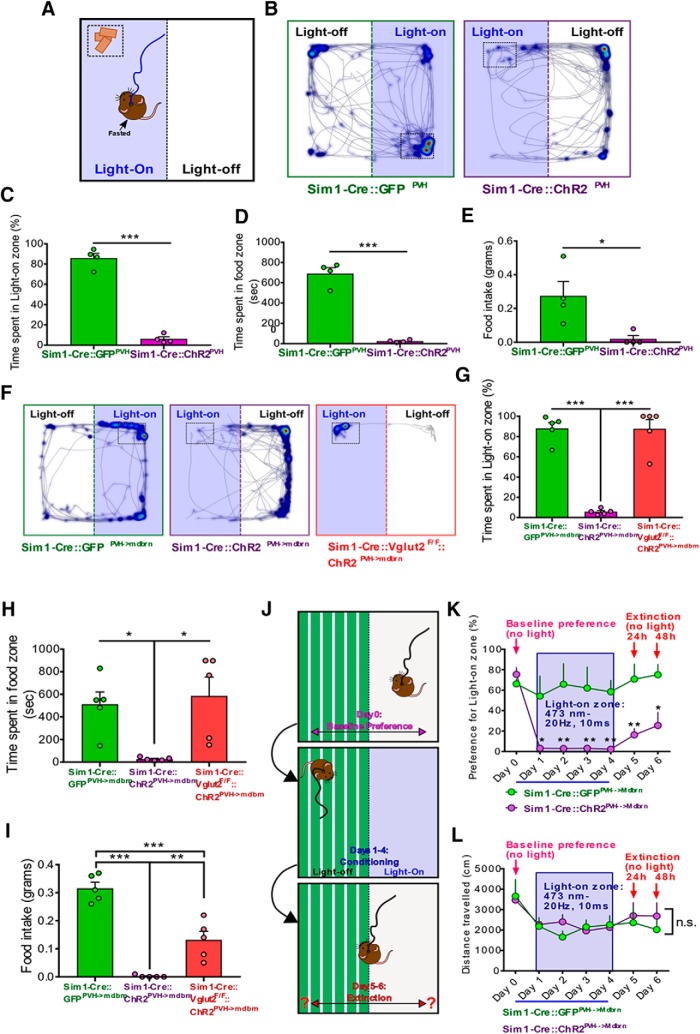
PVH→vMB activation on fast-refeeding and aversive conditioning. ***A***, Schematic of fast-refeeding experiment where food is placed in a corner of the light-paired side of the arena. ***B***, Representative heatmaps of time spent in arena location superimposed over tracks during fast-refeeding assay. Dashed rectangular area denotes food zone. One side of the chamber was paired with PVH photostimulation. ***C***, Percentage of total testing time spent in light-on zone during fast-refeeding assay; *n* = 4 animals/group. Unpaired *t* test: *t* = 15.17 df = 6; ***p < 0.0001. Error bars represent SEM. ***D***, Time spent in food zone during fast-refeeding assay; *n* = 4 animals/group. Unpaired *t* test: *t* = 11.4 df = 6; ***p < 0.0001. Error bars represent SEM. ***E***, Amount of food eaten during fast-refeeding assay; *n* = 4 animals/group. Unpaired *t* test: *t* = 2.936 df = 6; *p = 0.0261. Error bars represent SEM. ***F***, Representative heatmaps and activity tracks during fast-refeeding assay, where one side of the chamber containing food was paired with PVH→vMB (mdbrn) photostimulation. Dashed rectangular area denotes food zone. ***G***, Percentage of time spent in light-on zone during fast-refeeding assay for PVH→vMB photostimulation; *n* = 5 animals/group. One-way ANOVA, followed by Tukey’s multiple comparisons test: *F*_(2,12)_ = 57.5, *p* < 0.0001. Sim1-Cre::GFP^PVH->mdbrn^ versus Sim1-Cre::ChR2^PVH->mdbrn^, ***p < 0.0001; Sim1-Cre::ChR2^PVH->mdbrn^ versus Sim1-Cre::Vglut2^flox/flox^::ChR2^PVH->mdbrn^, ***p < 0.0001. Error bars represent SEM. ***H***, Time spent in food zone during fast-refeeding assay; *n* = 5 animals/group. One-way ANOVA, followed by Tukey’s multiple comparisons test: *F*_(2,12)_ = 6.952, *p* = 0.0099. Sim1-Cre::GFP^PVH->mdbrn^ versus Sim1-Cre::ChR2^PVH->mdbrn^, *p = 0.0289; Sim1-Cre::ChR2^PVH->mdbrn^ versus Sim1-Cre::Vglut2^flox/flox^::ChR2^PVH->mdbrn^, *p = 0.0127. Error bars represent SEM. ***I***, Amount of food eaten during the same assay; *n* = 5 animals/group. One-way ANOVA, followed by Tukey’s multiple comparisons test: *F*_(2,12)_ = 53.19, *p* < 0.0001. Sim1-Cre::GFP^PVH->mdbrn^ versus Sim1-Cre::ChR2^PVH->mdbrn^, ***p < 0.0001; Sim1-Cre::GFP^PVH->mdbrn^ versus Sim1-Cre::Vglut2^flox/flox^::ChR2^PVH->mdbrn^, ***p = 0.0002; Sim1-Cre::ChR2^PVH->mdbrn^ versus Sim1-Cre::Vglut2^flox/flox^::ChR2^PVH->mdbrn^, **p = 0.0030. Error bars represent SEM. ***J***, Schematic timeline showing experimental conditions during days of aversive conditioning and extinction tests. The initially most preferred side of a chamber containing contextual flooring cues was paired with PVH→vMB photostimulation on the subsequent days of conditioning. ***K***, Preference for light-paired side across conditioning days and extinction; *n* = 4–5 animals/group. Two-way repeated measures ANOVA, followed by Sidak’s multiple comparisons test: interaction *F*_(6,42)_ = 3.435, *p* = 0.0075; genotype *F*_(6,42)_ = 5.013, *p* = 0.0006; days of conditioning *F*_(1,7)_ = 23.73, *p* = 0.0018; subjects (matching) *F*_(7,42)_ = 3.496, *p* = 0.0048. Sim1-Cre::GFP^PVH->mdbrn^ versus Sim1-Cre::ChR2^PVH->mdbrn^: *p < 0.05; **p < 0.005. Error bars represent SEM. ***L***, Distance traveled during the conditioning assay for each day; *n* = 4–5 animals/group. Two-way repeated measures ANOVA: interaction *F*_(6,42)_ = 0.682, *p* = 0.6650; genotype *F*_(6,42)_ = 4.827, *p* = 0.0008; days of conditioning *F*_(1,7)_ = 0.09281, *p* = 0.7695; subjects (matching) *F*_(7,42)_ = 11.58, *p* < 0.0001. Error bars represent SEM.

We previously demonstrated that photostimulation of PVH neurons abruptly halts ongoing feeding, and in turn promotes repetitive grooming, a phenomenon that was reversible on light termination (Mangieri et al., 2018). To examine whether place avoidance elicited by PVH neuron activation would alter feeding, we modified the RTPP/A assay by placing food in a corner of the light-paired side of the arena (Fig. 5*A*). Following 24-h fast, GFP control mice approached the light-paired side of the chamber and proceeded to consume the food (Fig. 5*B–D*). In contrast, ChR2 mice attempted to approach the food zone, but spent significantly less time in the light-on side and food zone (Fig. 5*B*, right panel, *C*,*D*), and consequently ate significantly less than controls (Fig. 5*E*). Given that total locomotion during the assay were unchanged between groups, together, these data suggest that negative valence triggered by PVH photostimulation was sufficient to repel mice from an extremely salient goal, i.e., re-feeding after a long fast. We next performed the same assay on mice with photostimulation of local PVH fibers in vMB, and similarly found that Sim1-Cre::ChR2^PVH->vMB^ mice spent significantly less time in the light-paired side and food zone (Fig. 5*F–H*), and consumed significantly less (Fig. 5*I*). On the other hand, upon locating food, KO mice with the same stimulation tended to remain in the light-paired side (Fig. 5*F*), and spent a similar amount of time in the light-on side and food zone as GFP controls (Fig. 5*G*,*H*). The food intake in KO mice was more than that in the ChR2 group, but significantly less than GFP controls (Fig. 5*I*). The total distance traveled during the assay was unchanged between GFP control and Sim1-Cre::ChR1^PVH->vMB^ mice, but was significantly reduced in KO mice (data not shown). These data suggest a role for both glutamatergic and non-glutamatergic transmission in mediating the PVH→vMB circuit on feeding and locomotion during the fasted state.

Given the known role for the midbrain in learning, we next tested whether the aversion associated with light stimulation of PVH→midbrain could be learned. Sim1-Cre::ChR1^PVH->vMB^ and GFP control mice were conditioned across several consecutive days by pairing a previously preferred side of a chamber with light stimulation (Fig. 5*J*). As expected, Sim1-Cre::ChR1^PVH->vMB^ mice avoided the light-paired side during the 4 d of conditioning, spending significantly less time in that side (Fig. 5*K*). Interestingly, Sim1-Cre::ChR1^PVH->vMB^ mice persisted in avoiding the light-paired side during 24-h and 48-h extinction tests, when light was no longer applied (Fig. 5*K*). Day-to-day changes in locomotion during the entire testing session was unchanged between groups (Fig. 5*L*). Thus, the PVH→vMB circuit promotes a learned aversion process.

### Glutamatergic midbrain neurons are activated by PVH projections to drive escape behavior

**Figure 6.**
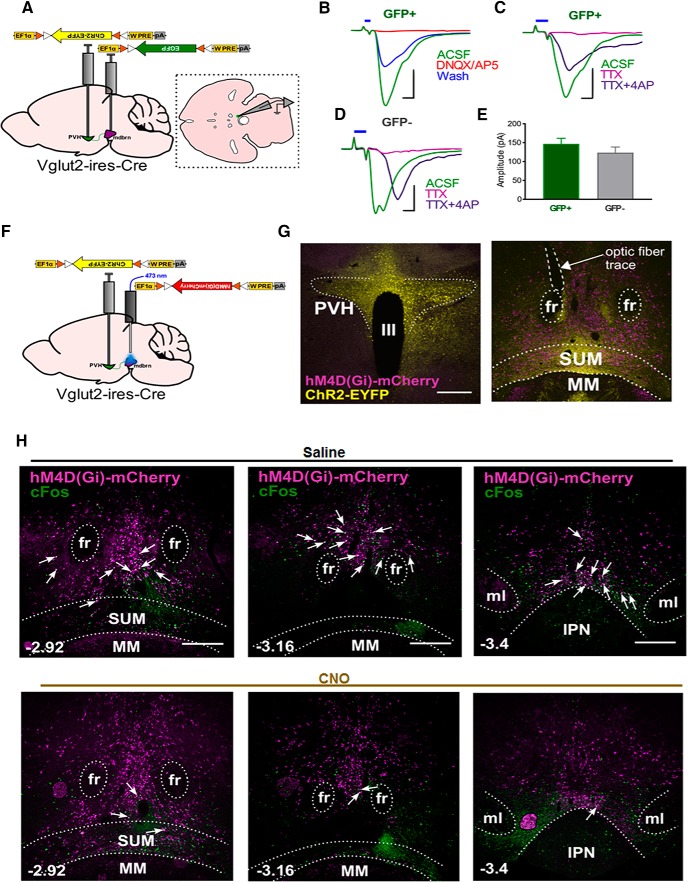
Activation of glutamatergic vMB neurons in escape behaviors. ***A***, Schematic showing viral delivery of ChR2-EYFP to PVH and EGFP to the midbrain in Vglut2-ires-Cre mice. Inset shows schematic of horizontal slice recordings in midbrain area. ***B***, Light-evoked excitatory postsynaptic responses (oEPSCs) in 20/20 GFP+ cells receiving input from PVH. Red traces showing oEPSCs blocked by glutamate receptor blockers, and partial recovery after wash-out of drugs (blue traces). Scale bar: 100 pA (vertical) and 2 ms (horizontal). ***C***, Light-evoked oEPSCs are blocked by TTX and recovered with addition of 4-AP in GFP+ cells. Scale bar: 100 pA (vertical) and 2 ms (horizontal). ***D***, Light-evoked oEPSCs are blocked by TTX and recovered with addition of 4-AP in GFP- cells. Scale bar: 50 pA (vertical) and 2 ms (horizontal). ***E***, Comparison of averaged oEPSC amplitude in GFP+ and GFP- cells. Unpaired *t* test: *t* = 0.9975 df = 36; *p* = 0.3252. Error bars represent SEM. ***F***, Experimental schematic for inhibiting midbrain glutamatergic neurons, using hM4D(Gi), concurrently with photostimulation of the PVH→vMB (mdbrn) circuit with ChR2. ***G***, Brain slice images of Vglut2-ires-Cre mice showing ChR2-EYFP expression in PVH (left), and hM4D(Gi)-mCherry expression in midbrain area (right). Dashed area shows optic fiber implant trace. III, third ventricle; fr, fasciculus retroflexus; MM, medial mammillary nucleus. Scale bar, 300 µm. ***H***, cFos immunostaining in the midbrain following intraperitoneal injection of saline (top images) versus CNO (bottom images) before PVH→vMB photostimulation in Vglut2-ires-Cre mice expressing inhibitory DREADD (hM4D(Gi)-mCherry) in midbrain at the bregma levels of –2.92 mm (left panels), –3.16 mm (middle panels), and –3.4 mm (right panels). Arrows point to mCherry-positive/cFos overlapping cells. Images representative of two animals per group. Scale bars, 300 µm. fr, fasciculus retroflexus; IPN, interpeduncular nucleus; ml, medial lemiscus; MM, medial mammillary nucleus.

Previous studies reported that glutamatergic neurons in vMB respond to aversive cues (Root et al., 2018), and their projections to the nucleus accumbens and lateral habenula drive aversion (Root et al., 2014; Qi et al., 2016). Since these glutamatergic neurons are located in the same region that receives dense PVH projections, i.e., the area medial to the VTA, we wondered whether PVH projections target glutamatergic neurons in the midbrain to promote aversion and escape behaviors. Since Sim1-Cre co-localizes with the majority of Vglut2 neurons in the PVH (Xu et al., 2013), we used Vglut2-ires-Cre mouse model (Vong et al., 2011) to target PVH neurons. To determine circuit connectivity, we delivered AAV-FLEX-ChR2-EYFP viruses to the PVH, and AAV-FLEX-GFP to vMB to visualize glutamatergic neurons. We performed whole-cell recordings on glutamatergic neurons in vMB, while photostimulating local PVH-Vglut2 fibers expressing ChR2 (Fig. 6*A*). We found that all GFP+ neurons patched showed EPSCs (oEPSCs; Fig. 6*B*). The currents could be blocked by bath application of tetrodotoxin (TTX), and subsequently rescued by 4-aminopyridine (4-AP), suggesting monosynaptic connectivity (Fig. 6*C*). We noted that the majority of GFP– cells patched (18/20) also received monosynaptic excitatory input from PVH (Fig. 6*D*), with a comparable latency and amplitude to GFP+ cells (Fig. 6*E*), suggesting diffusive innervation of PVH fibers onto vMB neurons. To examine the function of glutamatergic midbrain neurons, we silenced them before photostimulation by administrating CNO in Vglut2-ires-Cre mice injected with AAV-FLEX-hM4D(Gi)-mCherry virus into vMB and AAV-FLEX-ChR2-EYFP into the PVH (Fig. 4*F*,*G*). Photostimlation of PVH→vMB fibers produced cFos expression in vMB, many of which were found in glutamatergic neurons (Fig. 6*H*, top panels). Injection of CNO before PVH→midbrain photostimulation reduced cFos expression in vMB (Fig. 6*H*, bottom panels), suggesting effective CNO-induced inhibition of vMB glutamatergic neurons.

**Figure 7.**
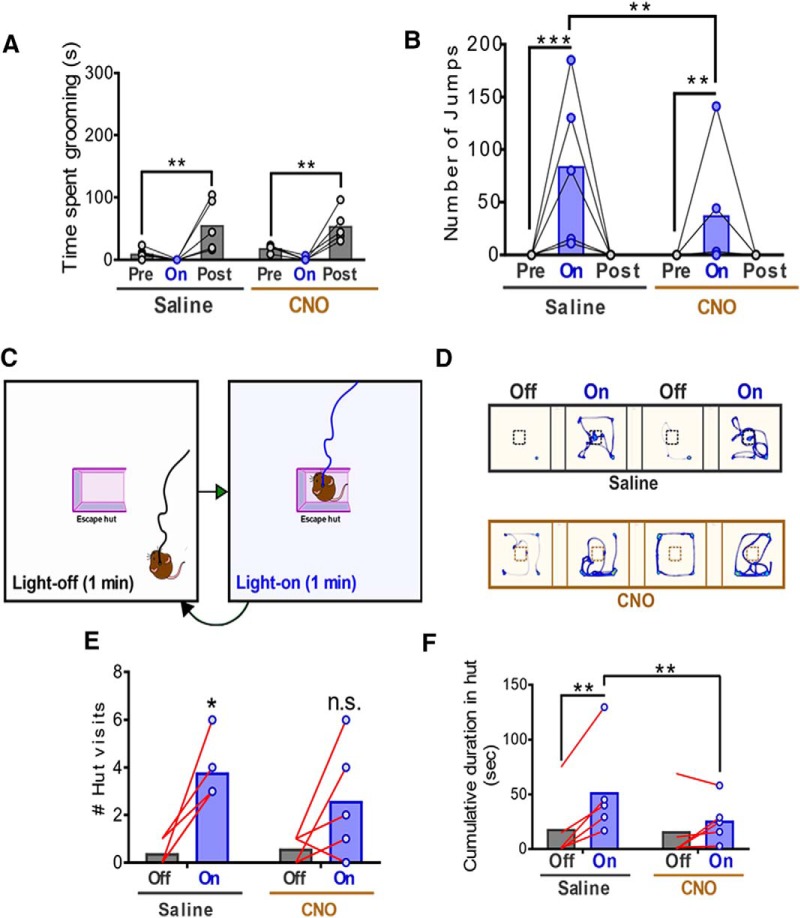
Activation of glutamatergic vMB neurons in escape behaviors. ***A***, Time spent grooming before, during, and after photostimulation of PVH→vMB (mdbrn) following intraperitoneal injection of saline or CNO; *n* = 5 animals. Two-way repeated measures ANOVA, followed by Dunnett’s multiple comparisons test: Interaction (drug × light epoch) *F*_(2,8)_ = 0.3522, *p* = 0.7135; drug *F*_(1,4)_ = 0.2098, *p* = 0.6707; light epoch *F*_(2,8)_ = 13.17, *p* = 0.0029. Pre-light versus Post-light, **p < 0.005. ***B***, Number of jumps counted during PVH→vMB photostimulation tests above, following intraperitoneal injection of saline or CNO; *n* = 5 animals. Two-way repeated measures ANOVA, followed by Dunnett’s and Sidak’s multiple comparisons tests: Interaction (drug × light epoch) *F*_(2,8)_ = 8.412, *p* = 0.0108; drug *F*_(1,4)_ = 8.412, *p* = 0.0441; light epoch *F*_(2,8)_ = 4.288, *p* = 0.0543. Pre-light versus light-on: **p < 0.005, ***p < 0.0005. Saline versus CNO: light-on, **p < 0.005. ***C***, Schematic for escape-hut assay. Light-off and light-on epochs alternated for 8 min. ***D***, Representative heatmap traces showing relative time spent in each arena location during the escape-hut assay. The first 4 min of the test is shown. ***E***, Quantification of the number of times animals visited the escape hut following intraperitoneal injection of saline or CNO. Number of hut visits were summed across four 1-min light-off periods and four 1-min light-on periods; *n* = 5 animals. Two-way repeated measures ANOVA, followed by Sidak’s multiple comparisons tests: Interaction (drug × light epoch) *F*_(1,4)_ = 1.054, *p* = 0.3627; drug *F*_(1,4)_ = 0.9091, *p* = 0.3943; light epoch *F*_(1,4)_ = 19.97, *p* = 0.0111. Off versus on, *p < 0.05. ***F***, Cumulative duration inside the hut during the escape-hut assay. Time spent inside the hut was summed across four 1-min light-off and four 1-min light-on epochs; *n* = 5 animals. Two-way repeated measures ANOVA, followed by Dunnett’s and Sidak’s multiple comparisons tests: interaction (drug × light epoch) *F*_(1,4)_ = 13.61, *p* = 0.0210; drug *F*_(1,4)_ = 0.4919, *p* = 0.5217; light epoch *F*_(1,4)_ = 11.49, *p* = 0.0275. Off versus on, **p < 0.005. Saline versus CNO: on, **p < 0.005.

Behaviorally, CNO administration in Vglut2-ires-Cre mice injected with AAV-FLEX-hM4D(Gi)-mCherry virus into vMB and AAV-FLEX-ChR2-EYFP into the PVH failed to affect self-grooming behavior (Fig. 7*A*), place avoidance (data not shown), or increased locomotion (data not shown) evoked by photostimulating the PVH→vMB circuit. However, CNO significantly reduced light-evoked escape jumping (Fig. 7*B*), indicating that vMB glutamatergic neurons play a significant role in escape, but not in other defensive behaviors evoked by light stimulation. A recent study showed that mice consistently and predictably return to a previously memorized shelter location upon experiencing threatening stimuli (Vale et al., 2017), so we next sought to explore the function of vMB glutamatergic neurons in this type of escape strategy. Toward this, we first injected Vglut2-ires-Cre mice with dual viral constructs as above, and then placed them in a testing chamber containing a shelter (escape hut) located in the middle of the arena (Fig. 7*C*). Mice were acclimated to the testing environment for 7 min to allow spontaneous discovery of the shelter (Vale et al., 2017), then were exposed to 1-min periods of no light, followed by 1 min intervals of light-on, repeated for 8 min (Fig. 7*C*). Light was pulsed at a lower frequency (5 Hz, 10 ms) during light-on periods to preclude potential interference from jumping activity. Remarkably, mice injected with saline before the trial consistently approached and hid in the shelter during the light-on epochs (Fig.7*D*, top panels, *F*). In contrast, although most mice injected with CNO approached the shelter during light-on periods (Fig. 7*E*) and displayed similar increases in locomotion on light stimulation during the assay (data not shown), they spent significantly less time hiding in the shelter (Fig. 7*D*, bottom panels, *F*). These findings provide further evidence that PVH projections onto midbrain glutamatergic neurons drive escape behaviors.

## Discussion

Threatening stimuli prompt a state of fear, leading to various defensive behavioral strategies, such as flight, avoidance, freezing, risk assessment and learned aversion, and compete with ongoing activities to promote survival (Blanchard and Blanchard, 2008). In this study, we describe a hypothalamic-vMB circuit engaged in triggering a classic set of emotional and behavioral aspects typical of defense. Notably, we found that the PVH→vMB connection drives acute and learned aversion, and is capable of increasing locomotion and escape behaviors. The aversive properties of PVH→vMB activation can override intrinsic homeostatic drive for feeding. From these results, we propose that the PVH→vMB circuit is part of the neural circuitry underlying behavioral and emotional processes that facilitate survival in a threatening situation.

We and others have previously shown that PVH neurons are directly involved in idiosyncratic behaviors, such as self-grooming, following stress (Füzesi et al., 2016; Mangieri et al., 2018). Our data here support the idea that the PVH signals negative valence, and is sufficient to produce stress-like and defensive responses. Given that repeated encounters with stressful situations may lead to a state of fear and avoidance (Steimer, 2002), it is not surprising that the same neural populations transmit interrelated messages. Although the hypothalamus has been previously regarded as a relay station for unconditioned defensive behaviors (Canteras, 2008), our study and others (Jennings et al., 2013a; Sternson, 2013; Kunwar et al., 2015) support the idea that discrete hypothalamic nuclei are sufficient for generating underlying emotional states concurrent with behavioral output. Given that subsets of PVH neurons drive the autonomic and neuroendocrine components of stress, the possibility of PVH neuron collaterals to brain sites that promote associated behaviors is becoming increasingly clear (Füzesi et al., 2016). Stress alters defensive expression (Mongeau et al., 2003; Li et al., 2018); therefore, neural circuits responsive to stress may modulate behavioral action based on context and/or experience. Supporting this, our findings show that the PVH→vMB circuit drives different defensive behaviors based on the testing environment. One striking finding in this study is that optogenetic stimulation of PVH→vMB projections drives aversion learning. This aversion learning may be related to a general function of vMB neurons, as supported by previous studies showing that distinct vMB circuits have been shown to drive emotional learning processes (Lammel et al., 2012; Root et al., 2014; Barbano et al., 2016; Nieh et al., 2016; Qi et al., 2016), and alter behavioral outputs in response to stress (Chaudhury et al., 2013; Tye et al., 2013). To our knowledge, this is the first study that links the PVH function to behavioral conditioning. Further studies are required to examine the circuit mechanisms underlying the aversion learning.

The majority of PVH neurons use glutamate as a neurotransmitter (Xu et al., 2013). Consistently, our results suggest that glutamatergic transmission from PVH onto vMB neurons was required for defensive behaviors, and contributed significantly to the evoked self-grooming behavior. Interestingly, glutamate release from PVH neurons was not absolutely required for the evoked grooming response, suggesting non-glutamatergic, likely neuropeptidergic action, which is in stark contrast to an absolute requirement for glutamate release from lateral hypothalamic neurons in promoting self-grooming behaviors (Mangieri et al., 2018). Of note, less time spent engaged in grooming behavior in the absence of glutamate release is likely due to the competing time spent in jumping behavior, which is supported by increased grooming post-stimulation when no jumping was observed (Fig. 4*B*). Delayed postsynaptic responses inherent to neuropeptide signaling (van den Pol, 2012) may indeed explain the persistence in grooming following light cessation, as well as the delayed initiation of grooming following light stimulation of PVH in the absence of glutamate release. Provided that activation of corticotropin-releasing hormone (CRH) cells in PVH promotes grooming (Füzesi et al., 2016), and that VTA neurons expressing CRH receptors play a role in stress-induced alterations in behavior (Holly et al., 2016), it is possible that CRH signaling from PVH onto midbrain neurons drives the evoked grooming behavior observed here. Nevertheless, future investigation will be needed to identify the specific neuropeptide populations involved.

Despite extensive research on the impact on behavior via changes in vMB neuron activity, specific upstream sites for glutamatergic transmission onto ventral midbrain glutamate neurons remain largely unexplored (Morales and Margolis, 2017). Here, we identified the PVH as a source of glutamatergic input onto ventral midbrain neurons in driving defensive behaviors. Given the previous findings on vMB glutamatergic neurons in aversion (Root et al., 2014; Qi et al., 2016), we explored the contribution of these neurons. We found that neurons indeed contributed to the evoked escape behaviors, as evidenced from reduced jumping and hiding behavior when vMB glutamatergic neurons were silenced. However, glutamatergic neural silencing failed to reduce time spent in grooming or completely suppress light-evoked escape behaviors. One of potential underlying reasons may be due to inherent caveats of less than one hundred percentage transfection with hM4D-Gi via viral targeting; thus, incomplete silencing of glutamatergic neurons may have insufficiently precluded escape responses. Alternatively, given the relatively mild effect on behaviors by silencing glutamatergic neurons, it is most likely that non-glutamatergic midbrain neurons also contribute significantly to mediating the behavioral output of the PVH projection. Notably, GABA-releasing VTA neurons are a good candidate for future interrogation, as they have been shown to respond to aversive stimuli (Tan et al., 2012) and drive aversion processes (Tan et al., 2012; van Zessen et al., 2012). In addition, although our data suggest that only a small number of dopamine neurons are activated by PVH inputs, given their known role in behavior, it is possible dopamine cells may also contribute to aversive properties (Lammel et al., 2012) and increased locomotion (Boekhoudt et al., 2016) in response to PVH neuron activation.

The PVH sends outputs to several brainstem regions, some of which have been implicated in various defensive behaviors. For example, the PAG has been shown to participate in freezing, flight, and avoidance behaviors (Deng et al., 2016; Tovote et al., 2016). Recently, a specific neural subset in the PBN, a major relay for sensory information, was implicated in defensive expression following recall of fearful memories (Campos et al., 2018). Both PAG and PBN are known downstream sites for the PVH in feeding regulation (Stachniak et al., 2014; Garfield et al., 2015). Since the PVH to vMB projection promotes defensive behaviors, it is conceivable that PVH projections to the PAG and PBN may also exert a similar function. Notably, given an incomplete reversal in behavioral phenotypes by either vMB glutamate receptor antagonism or inhibition of midbrain glutamatergic neurons, it is possible that PVH-collateral fiber activation, due to back propagation, may have resulted in activation of PAG and/or PBN. Ultimately, this may lead to redundant manifestation of the observed residual defensive behaviors. This possibility is supported by the similar effect on suppressing feeding by PVH projections to vMB (this study), PAG, and PBN (Stachniak et al., 2014; Garfield et al., 2015). Future functional tracing and behavioral studies will help delineate how distinct PVH projections are coordinated in the generation of defense and feeding in response to changing environments.

Defensive behaviors such as shelter-seeking and escape represent innate behavioral components and are crucial for survival. Maladaptive coping strategies in people, such as social avoidance and behavioral compulsions, may be illustrative of hardwired responses gone awry in a modern world posing an onslaught of novel environmental challenges. Thus, it has become increasingly important to investigate the neural basis of such behaviors, as they can often lead to paralyzing mental disorders such as generalized anxiety (Kashdan et al., 2006). Here, we have uncovered a hypothalamic-vMB pathway that drives and/or promotes conditioned aversion and escape, adding to the accumulating picture of how the brain integrates and produces emotions and behaviors underlying adaptive, and possibly maladaptive, strategies for survival.

## Synthesis

Reviewing Editor: Quentin Pittman, University of Calgary Decisions are customarily a result of the Reviewing Editor and the peer reviewers coming together and discussing their recommendations until a consensus is reached. When revisions are invited, a fact-based synthesis statement explaining their decision and outlining what is needed to prepare a revision will be listed below. Note: If this manuscript was transferred from JNeurosci and a decision was made to accept the manuscript without peer review, a brief statement to this effect will instead be what is listed below.

The great majority of the comments from the JNS reviews were for clarification/corrections. These have been adequately dealt . A lingering concern is that there was non-specific activation of all PVN neurons , rather than phenotypically distinct ones ( eg CRH, TRH OXT, AVP etc).However, there is some value in the identification of the pathway and the pharmacological and slice experiments indicate a glutamatergic component. Furthermore, it is recognized that there is a considerable body of work presented here.
